# Ocular Toxicity Associated with Chlorpromazine: A Case Report

**DOI:** 10.1192/j.eurpsy.2026.11536

**Published:** 2026-07-31

**Authors:** S. Ben Abderrahmen, A. Ghorbel

**Affiliations:** 1 Psychiatry; 2 Ophtalmology, CHU Farhat Hached, Sousse, Tunisia

## Abstract

**Introduction:**

Chlorpromazine is a first-generation antipsychotic first-generation antipsychotic of the phenothiazines is widely used in psychiatry. Several side side effects have been reported due to its anti-dopaminergic and anticholinergic properties.

**Objectives:**

We report a clinical case of ocular toxicity in a patient on chlorpromazine and discuss potential pathophysiological mechanisms.

**Methods:**

Mr. C.A., aged 36, treated for 19 years for resistant schizophrenia, complains of bilateral visual blur associated with a bilateral decrease in visual acuity which has been evolving for a year. He has been on clozapine 800 mg/d for 6 years and L-thyroxine 125mg/d since then. He was also put on chlopromazine 300 mg/d for 10 years, a treatment which was stopped 2 years ago. Ophthalmological examination showed corrected visual acuity: 8/10 in the right eye and 7/10 in the left, as well as slate-blue pigmentation of the conjunctiva, sparse whitish endothelial corneal deposits and yellowish star-shaped deposits on the anterior surface of the anterior crystalloid. Fundus examination was normal. Chlorpromazine was considered to be a drug of abuse, given its potential ocular toxicity, the association of conjunctival pigmentation, white endothelial deposits and star-shaped crystallites, and the exclusion of other possible causes, particularly iatrogenic ones such as prolonged use of corticoids, fluoroquinolones and amiodarone.

**Results:**

This case highlights the potential ocular toxicity of chlorpromazine. Prolonged use is associated with pigmentary changes in sun-exposed areas. The photo-toxic action of light on high quantities of phenothiazines provokes an inflammatory reaction followed by increased melanin production. This melanin-chlorpromazine bond causes slate-blue discoloration of the conjunctiva and sclera, corneal pigment deposits (white, yellow, brown or black), stellate and polar cataracts. Other effects, such as accommodation disorders and dry eyes, are linked to the anticholinergic effect.

Prolonged use of phenothiazines (over 20 months), especially at high doses (>800 mg/d), requires regular ophthalmological monitoring, even in the absence of symptoms, due to the risk of pigmentary deposits. If visual acuity is unaffected, treatment can be continued, as reduction or discontinuation does not cause lesions to disappear.

**Conclusions:**

This case highlights the need for regular ophthalmological monitoring of patients undergoing treatment with phenothiazines, such as chlorpromazine, due to the risk of pigmentary deposits in the cornea and lens, even in the absence of symptoms.

**Disclosure of Interest:**

None Declared

